# Cell Migration in the Immune System: the Evolving Inter-Related Roles of Adhesion Molecules and Proteinases

**DOI:** 10.1155/2000/79045

**Published:** 2000

**Authors:** Joseph A. Madri, Donnasue Graesser

**Affiliations:** 1 Department of Pathology Yale University New Haven CT 06510 USA; 2 Department of lmmunobiology Yale University New Haven CT 06510 USA; 3 Department of Pathology Yale University School of Medicine 310 Cedar Street New Haven CT 06510 USA

**Keywords:** T lymphocyte, endothelial cell, matrix metalloproteinase, inflammation, transendothelial migration, integrins, cell adhesion molecules

## Abstract

Leukocyte extravasation into perivascular tissue during inflammation and lymphocyte homing to lymphoid organs involve transient adhesion to the vessel endothelium, followed by transmigration through the endothelial cell (EC) layer and establishment of residency at the tissue site for a period of time. In these processes, leukocytes undergo multiple attachments to, and detachments from, the vessel-lining endothelial cells, prior to transendothelial cell migration. Transmigrating leukocytes must traverse a subendothelial basement membrane en route to perivascular tissues and utilize enzymes known as matrix metalloproteinases to make selective clips in the extracellular matrix components of the basement membrane. This review will focus on the evidence for a link between adhesion of leukocytes to endothelial cells, the induction of matrix metalloproteinases mediated by engagement of adhesion receptors on leukocytes, and the ability to utilize these matrix metalloproteinases to facilitate leukocyte invasion of tissues. Leukocytes with invasive phenotypes express high levels of MMPs, and expression of MMPs enhances the migratory and invasive properties of these cells. Furthermore, MMPs may be used by lymphocytes to proteolytically cleave molecules such as adhesion receptors and membrane bound cytokines, increasing their efficiency in the immune response. Engagement of leukocyte adhesion receptors may modulate adhesive (modulation of integrin affinities and expression), synthetic (proteinase induction and activation), and surface organization (clustering of proteolyric complexes) behaviors of invasive leukocytes. Elucidation of these pathways will lead to better understanding of controlling mechanisms in order to develop rational therapeutic approaches in the areas of inflammation and autoimmunity.


*Developmental Immunology*, 2000, Vol. 7(2-4), pp. 103-116
Reprints available directly from the publisher
Photocopying permitted by license only

© 2000 OPA (Overseas Publishers Association)
N.V. Published by license under the
Harwood Academic Publishers imprint,
part of the Gordon and Breach Publishing Group.
Printed in Malaysia

### Cell Migration in the Immune System: the Evolving Inter-Related Roles of Adhesion Molecules and Proteinases*

JOSEPH A. MADRI[a†] and DONNASUE GRAESSER[ab]

[a]*Departments of Pathology and* [b]*Immunobiology, Yale University New Haven, CT 06510*

Leukocyte extravasation into perivascular tissue during inflammation and lymphocyte homing to lymphoid organs involve transient adhesion to the vessel endothelium, followed by transmigration through the endothelial cell (EC) layer and establishment of residency at the tissue site for a period of time. In these processes, leukocytes undergo multiple attachments to, and detachments from, the vessel-lining endothelial cells, prior to transendothelial cell migration. Transmigrating leukocytes must traverse a subendothelial basement membrane en route to perivascular tissues and utilize enzymes known as matrix metalloproteinases to make selective clips in the extracellular matrix components of the basement membrane. This review will focus on the evidence for a link between adhesion of leukocytes to endothelial cells, the induction of matrix metalloproteinases mediated by engagement of adhesion receptors on leukocytes, and the ability to utilize these matrix metalloproteinases to facilitate leukocyte invasion of tissues. Leukocytes with invasive phenotypes express high levels of MMPs, and expression of MMPs enhances the migratory and invasive properties of these cells. Furthermore, MMPs may be used by lymphocytes to proteolytically cleave molecules such as adhesion receptors and membrane bound cytokines, increasing their efficiency in the immune response. Engagement of leukocyte adhesion receptors may modulate adhesive (modulation of integrin affinities and expression), synthetic (proteinase induction and activation), and surface organization (clustering of proteolytic complexes) behaviors of invasive leukocytes. Elucidation of these pathways will lead to better understanding of controlling mechanisms in order to develop rational therapeutic approaches in the areas of inflammation and autoimmunity.

*Keywords:* T lymphocyte, endothelial cell, matrix metalloproteinase, inflammation, transendothelial migration, integrins, cell adhesion molecules

### INTRODUCTION

### Leukocyte Extravasation

During the processes of homing and extravasation at sites of inflammation, circulating leukocytes become tissue-resident cells for variable periods of time. The transmigration of leukocytes from the bloodstream into perivascular tissue is a critical event in both these processes. During homing, lymphocytes bind to adhesion receptors displayed on high endothelial venules (HEV) as points of entry into secondary lymphoid

* Supported, in Part by USPHS Grant RO1-HL-51018 to JAM.
† All correspondence to: Joseph A. Madri, Ph.D., M.D., Department of Pathology, Yale University School of Medicine, 310 Cedar Street, New Haven, CT 06510. TEL: 203–785–2763, FAX: 203–785–7373.

organs (except spleen where lymphocytes enter via blood sinusoids in the marginal zones). Granulocytes and monocytes do not home but, along with lymphocytes, will adhere to the microvascular endothelium in inflamed tissues, and in response to chemoattractant or adhesive gradients, migrate across the endothelial cell layer and accumulate at the site of injury or infection. Both the processes of homing and extravasation during inflammation are dynamic, involving multiple attachments to, and detachments from, a variety of cell types and extracellular matrix components, before encountering antigen or antigen-presenting cells in the tissues and delivering an appropriate immunological response. The general mechanisms involved in both processes are similar (Reviewed in Springer, 1995a, 1995b). Briefly, the extravasating leukocytes will first be tethered to the endothelial cells, roll along the blood vessel wall, integrins on the cell surface will be activated and used to adhere tightly to the endothelial cells, leukocytes will transmigrate across the endothelial layer, traverse the subendothelial basement membrane, and finally migrate into the perivascular tissue. All these steps are mediated by binding of adhesion molecules on the extravasating leukocytes to counter-receptors on endothelial cells lining the vessel wall.

Initial tethering and rolling of leukocytes on the vessel wall are mediated by a class of molecules known as selectins, which form labile adhesions that permit leukocytes to subsequently roll in the direction of flow (Lawrence and Springer, 1991; Lawrence and Springer, 1993; vonAndrian, 1991). Selectins appear to recognize sialylated carbohydrate determinants on their counter-receptors (Berg et al., 1991; Lasky, 1992; Rosen, 1993). L-selectin (LAM-1, MEL-14, Leu-8) is expressed on all circulating leukocytes, except for a subpopulation of lymphocytes (Gallatin et al., 1983; Kansas et al., 1985; Lewinsohn et al., 1987) and recognizes at least 2 mucin-like molecules on HEV, GlyCAM-1 (Lasky et al., 1992) and CD34 (Baumhueter et al., 1993, 1994). CD34 is also expressed on capillaries but is absent from most large vessels (Fina et al., 1990). Conversely, leukocytes can bind to E-selectin (ELAM-1) or P-selectin expressed on endothelial cells at inflammatory sites. E-selectin is induced on microvascular endothelial cells in response to cytokines such as IL-1, LPS, or TNF-α (Bevilacqua et al., 1987). Endothelial cell P-selectin is stored in Weibel Palade bodies and rapidly mobilized to the plasma membrane to bind neutrophils and monocytes in response to mediators of acute inflammation (Geng et al., 1990; Larsen et al., 1989; McEver et al., 1989). E- and P-selectin recognize distinct, but closely related ligand structures (Alon et al., 1995; Larsen et al., 1992). A rapid selectin-ligand association rate facilitates the initial tethering, and a rapid dissociation rate allows the leukocyte to roll forward (Lawrence and Springer, 1991; Hammer and Apte, 1992).

Following rolling, given the appropriate signals, some leukocytes will adhere tightly to the endothelium via interactions between molecules of the immunoglobulin super-family (on the endothelial cells) and integrins (on the leukocytes). The integrin family of glycoproteins consists of more than 20 non-covalently bound αβ heterodimers and bind to a diverse array of ligands, including extracellular matrix proteins, plasma proteins that are deposited at sites of injury, such as fibrinogen, and receptors on other cells (Hemler, 1990; Hynes, 1992). ICAM-1, −2, and −3 (Intercellular Adhesion Molecule, CD54, CD102, and CD50) on endothelial cells binds to LFA-1 (Leukocyte Function-associated Antigen, αLβ2 integrin, CD11a/CD18) on T cells, B cells, monocytes and neutrophils (deFourgerolles and Springer, 1992; Marlin and Springer, 1987; Rothlein et al., 1986; Shimizu et al., 1991; Staunton et al., 1989) as well as Mac-1 (αMβ2 integrin, CD11b/CD18) on neutrophils and monocytes (Diamond et al., 1990, 1991; Kishimoto et al., 1989). VCAM-1 (Vascular Cell Adhesion Molecule, CD106) on endothelial cells binds both VLA-4 (α4β1 integrin, CD49d/CD29) and α4β7 integrin on leukocytes (Chan et al., 1992; Elices et al., 1990; Osborne et al., 1989; Ruegg et al., 1992; Shimizu et al., 1991; Vennegoor et al., 1992). VLA-4 integrin also binds to the alternatively spliced CS-1 peptide of the extracellular matrix glycoprotein fibronectin (Wayner et al., 1989). In addition, during lymphocyte recirculation to mucosal tissues, the mucosal addressin, MAdCAM-1, on Peyer's patch HEV binds α4β7, but not α4β1, integrin (Berlin et al., 1993; Hu et al., 1992; Streeter et al., 1988). Another member of the immunoglobulin super-family, PECAM-1 (Platelet Endothelial Cell Adhesion Molecule-1, CD31), has been localized on both T cells and endothelial cell surfaces (Newman et al., 1990; Albelda et al., 1990, 1991) and is thought to interact in a homophilic manner during T cell-endothelial cell interactions (Bogen et al., 1992). PECAM-1 has been shown to participate in transendothelial migration and migration through extracellular tissues by neutrophils, natural killer cells, and monocytes (Berman et al., 1996; Christofidou-Solomidou et al., 1997; Liao et al., 1995; Liao et al., 1997; Muller, 1995).

Integrins must be activated in order to bind to the appropriate ligands (Diamond and Springer, 1994). Activation may be regulated by a number of extracellular signals including cross-linking of the antigen receptor, phorbol esters, binding to complement components or to fibrinogen, or other cell-cell adhesive interactions (Arroyo et al., 1993; Berman and Muller, 1995; Berman et al., 1996; Smith et al., 1989; Tanaka et al., 1992; Wilkins et al., 1991). During rolling, leukocytes come into contact with chemoattractants on the microvascular endothelium, which bind G-protein coupled receptors on leukocytes (Miller and Krangel, 1992; Springer, 1995a, 1995b) and stimulate changes such as degranulation, shape change, actin polymerization, and respiratory burst, as well as activating the adhesiveness of integrin molecules (Lo et al., 1989; Smith et al., 1991). Adhesiveness of integrin molecules such as Mac-1 and LFA-1 on neutrophils and monocytes is activated by chemokines including N-formylated peptide and IL-8. Increased adhesiveness is not due to changes in expression, but is likely due to conformational changes that increase their affinity for endothelial cell ligands (Diamond and Springer, 1994; Landis et al., 1993; Lollo et al., 1994). Integrin adhesiveness is transient and is down-regulated within minutes (Dustin and Springer, 1989; Lo et al., 1989). This cycling between adhesive and non-adhesive states allows the cells to rapidly regulate adhesion to ligands on opposing cell surfaces and matrices (Diamond and Springer, 1994).

Extravasating leukocytes then need to release adhesive interactions with the luminal surface of the blood vessel wall and migrate across the endothelium where they encounter a complex, organized extracellular matrix (ECM) comprised of several collagenous and non-collagenous components as they migrate through the basement membrane and into the interstitial matrix (Shimizu & Shaw, 1991). The movement of leukocytes across the endothelium and into the ECM is a dynamic process in which both leukocytes and endothelial cells actively modulate their cell-cell and cell-ECM interactions (Madri et al., 1996). Leukocyte migration through the ECM is mediated by a number of parameters including integrin surface expression (Graesser et al., 1998; Juliano & Haskill, 1993; Leavesly et al., 1994; Romanic & Madri, 1994; Romanic et al., 1997; Shimizu & Shaw, 1991); integrin affinity (Arroyo et al, 1993; Hourihan et al., 1993; Shimizu & Shaw, 1991; Smith et al., 1989; Wilkins et al., 1991); ECM synthesis and deposition (Hauser et al., 1993; Madri et al., 1988); and ECM degradation (Goetzl et al., 1996; Graesser et al., 1998; Leppert et al., 1995a, 1195b, 1996; Montgomery et al., 1993; Romanic & Madri, 1994). In this review we will discuss the contributions and inter-relationships of integrin engagement and matrix metalloproteinases in the transition of leukocytes, in particular T lymphocytes, from circulating cells to tissue resident cells.

### Matrix metalloproteinases in leukocytes

Even with the precise combination of adhesion molecules and chemokines, not all cells acquire a migratory phenotype and become invasive. Several studies have implicated MMPs as an important component in tumor cell migration and tissue invasion (Fessler et al., 1984; Höyhtyä et al., 1990; Liotta et al., 1979; Mignatti et al., 1986; Salo et al., 1983). It is hypothesized that, like metastasizing tumor cells, leukocytes utilize matrix metalloproteinases (MMPs) to degrade the extracellular matrix (ECM) components of the basement membrane during transmigration across blood vessel walls, and the regulation of expression and activation of MMPs may be critical for leukocyte transmigration (Goetzl et al., 1996; Leppert et al., 1995a, 1995b, 1996; Xia et al., 1996a, 1996b). Indeed, leukocytes that have infiltrated tissues or have acquired an invasive phenotype often have elevated expression of MMPs. Studies on cell lines from human patients with a number of lymphoproliferative diseases such as Burkitt's lymphoma, B-cell lymphoblastic leukemia, and multiple myeloma, have found that these "reactive" lymphoid cells express elevated levels of either MMP-2, MMP-9 or both (Stetler-Stevenson et al., 1997; Vacca et al., 1998). Some of these cell lines also express TIMP-1 or TIMP-2 (Tissue Inhibitors of Metalloproteinases), the physiologic inhibitors for MMP-9 and MMP-2, respectively. MMP-9 levels are increased in the cerebral spinal fluid of rabbits with induced bacterial meningitis, and this increase was found to be associated with leukocytes that had infiltrated the CNS (Azeh et al., 1998). In a study of human patients with viral meningitis, which is characterized by invasion of neutrophils, monocytes, and lymphocytes into the subarachnoidal space, MMP-2 levels were found to be elevated (Kolb et al., 1998). Recently, a novel MMP, MMP-19 has been isolated from the inflamed synovium of patients with rheumatoid arthritis (Sedlaceck et al., 1998). MMP-19 is also found on the surface of Jurkat lymphoma cells. MMP-2, MMP-9, and MMP-7 (matrilysin) have also been found to be up-regulated in macrophages within inflammatory multiple sclerosis lesions, and are believed to play a part in breakdown of the blood-brain barrier, leukocyte emigration, and tissue destruction in multiple sclerosis (Anthony et al., 1997).

In addition, studies in our laboratory and others have shown that MMPs play a role in experimental rodent models of multiple sclerosis (Anthony et al., 1997; Gijbels et al., 1994; Graesser et al., 1998; Hewson et al., 1995)

### Regulation of matrix metalloproteinases via integrin-mediated signals

The signals that regulate expression of MMPs in leukocytes are largely unknown, and are the subject of ongoing research. It is possible that signals mediated by the engagement of leukocyte adhesion receptors during the initial stages of transendothelial cell migration may lead to secretion or activation of MMPs in the migrating cells. In particular, engagement of integrin receptors is thought to trigger a number of intracellular signaling events in T cells (Graesser et al., 1998; Hynes, 1992; Romanic & Madri, 1994; Romanic et al., 1997; Shimizu And Shaw, 1991). For instance, engagement of VLA-4 either with antibodies to the α4 subunit or with the alternatively spliced CS1 domain of fibronectin has been shown to stimulate tyrosine phosphorylation of a 150 kDa protein in T cells (Nojima et al., 1992). Ligation of VLA-4 and VLA-5 (α5β1) integrins has been shown to act as a costimulus along with the engagement of CD3 to mediate T cell proliferation (Davis et al., 1990; Nojima et al., 1990). Engagement of VLA-4 integrin with VCAM-1 or CS1 mediates changes in the surface expression of VLA-4 and LFA-1 and in adhesion to several ECM components (Romanic & Madri, 1994; Romanic et al., 1997). In addition, engagement of the VLA-5 integrin on T cells with fibronectin has been shown to induce the expression of the AP-1 transcription factor which regulates IL-2 transcription (Yamada et al., 1991).

Recent data suggests that integrin-mediated signals may also regulate MMP secretion and/or activity in a number of cell types. For each cell type examined, there appears to be some degree of specificity, ie. only certain integrin engagements will induce particular MMPs. In several tumor cell models, integrins have been shown to play a role in transducing signals that either enhance the expression of matrix metalloproteinases, their inhibitors, or their activators (Remy et al., 1999). In human rhabdomyosarcoma cells, antibodies against α2 and α3 integrin subunits enhanced the secretion of MMP-2, activation of the enzyme, and subsequent invasion of matrigel by the cells (Kubota et al., 1998). Ligation of α6β1 integrin on a human melanoma cell line promoted invasiveness by causing a 2–3 fold increase in ECM degradation (Nakahara et al., 1996). The induced ECM degradation was associated with increased surface expression of a gelatinase enzyme, and localization of the gelatinase at invadopodia. In a similar assay, invasion of matrigel by human glioma cells was increased by treatment with antibodies against either α3β1 integrin or α5β1 integrin (Chintala et al., 1996), which also resulted in decreased expression of these integrins and increased expression of MMP-2. Engagement of α5β1 integrin by binding the RGD peptide fragment of fibronectin was found to induce stromelysin, collagenase, and MMP-9, and increase the expression of MMP-2 in rabbit articular chondrocytes (Arner and Tortorella, 1995). αvβ3 and α5β1 integrins have been shown to play a role in human melanoma cell invasion by regulating expression of MMP-2 (Seftor et al., 1992, 1993). Induction of MMPs via integrin-mediated signals has been observed in non-cancerous cells as well. Binding of chondrocytes to RGD peptide has been shown to induce the synthesis of stromelysin, MMP-1, MMP-9 and increase the expression of MMP-2. (Arner and Tortorella, 1995). In fibroblasts, the expression of several MMPs is regulated by adhesion to fibronectin (Huhtala et al., 1995; Tremble et al, 1995) and tenascin (Tremble et al., 1994). Additionally, recent studies in our lab have shown that MMPs induced via integrin-mediated signals play a role in both in vivo and in vitro models for T cell transmigration (Baron et al., 1993; Graesser et al., 1998; Romanic and Madri, 1994).

In addition to modulation of MMP induction, integrin engagement has been shown to affect the activation state of MMP-2. MT-1-MMP (membrane-type matrix metalloproteinase, MMP-14) is a transmembrane metalloproteinase that has been implicated as the molecule that both tethers and activates MMP-2 at the cell surface (Butler et al., 1998; Cao et al., 1995; Kinoshita et al., 1998; Sato, et al., 1994; Strongin et al., 1995). A tertiary complex is formed between MT1-MMP, MMP-2, and TIMP-2, which acts as a bridging molecule. In one study, the activity of MMP-2 was increased in human breast carcinoma cells upon adhesion to collagen I, but not collagen IV, laminin, or fibronectin (Thompson et al., 1994). The activation of MMP-2 in this model was inhibited by metalloproteinase inhibitors, suggesting that the activating enzyme is itself a metalloproteinase. In a second study of breast carcinoma cells, adhesion to SPARC/osteonectin and collagen was shown to induce MMP-2 activation in cells that expressed MT1-MMP (Gilles et al., 1998). Interestingly, MT1-MMP was not expressed on non-invasive breast cell counterparts, and MMP-2 activation was not induced upon adhesion. Simultaneous induction of MMP-2 and its activator MT1-MMP are noted during in vitro 3-dimensional collagen matrix culture of endothelial cells as an in vitro model of angiogenesis (Haas et al., 1998). In this model, MMP-2 activation is blocked with matrix metalloproteinase inhibitors, and subsequently endothelial cell tube-formation is arrested. Thus, the modulation of MMP activity by integrin appears to be regulated at several levels, including enzyme induction, enzyme activation and enzyme inhibition. This broad range of control mechanisms allow for a fine-tuned tightly regulated response during extravastion/invasion.

### INTEGRIN-MEDIATED REGULATION OF MMP-2 IN T LYMPHOCYTES

### A role in T cell transmigration in vivo?

MMPs have been shown to be regulated by integrin-mediated signals in infiltrating lymphocytes. Studies in our lab have shown that MMP-2 is induced or upregulated in specific T cell clones adherent to either recombinant VCAM-1, VCAM-1(+) endothelial cells, or the CS1 peptide of fibronectin (Graesser et al., 1998; Romanic and Madri, 1994). The induction or upregulation of MMP-2 is specifically mediated by α4 integrin, and is not seen in clones adherent to recombinant ICAM-1, VCAM-1(-) endothelial cells, or the RGD peptide of fibronectin. Furthermore, the T cell clones constitutively express the enzyme responsible for activating MMP-2 (MT1-MMP) as well as TIMP-2, which is the "bridging" molecule that tethers MMP-2 to cell surface MT1-MMP. In our studies, upregulation of MMP-2 on the T cell surface is always accompanied by coordinate activation of the enzyme.

Both in vivo and in vitro models have been utilized successfully to study the functional role of α4 integrin-mediated induction of MMP-2. Studies by our group and others (Baron et al., 1993; Graesser et al., 1998; Keszthelyi et al., 1996; Kuchroo et al.,

1991; Soilu-Hanninen et al., 1997; Yednock et al., 1992) strongly suggest that expression of VLA-4 (α4β1) integrin is an important determinant for T cell entry into the central nervous system (CNS) leading to the development of experimental autoimmune encephalomyelitis (EAE). EAE is a rodent model for the human disease multiple sclerosis, induced by immunization with peptides of myelin basic protein (MBP) or autoreactive MBP-specific CD4+ Th1 cells, characterized by invasion of the CNS by MBP-specific T cells and ascending paralysis of the animal. We have used a murine EAE model to study the transmigration of T lymphocytes from the blood into CNS parenchyma (Baron et al., 1993; Graesser et al., 1998) and have isolated and characterized several T cell clones having identical antigen specificity, proliferative capacity, and cytokine profiles, but differing in abilities to elicit disease in adoptive transfer studies. We have found that expression of α4 integrin was a major pathogenic factor; encephalitogenic clones expressed ten-fold more α4 integrin than non-encephalitogenic clones. α4 integrin high expressors entered brain parenchyma, while α4 integrin low or null expressing clones did not. Antibodies directed against either α4 integrin or its endothelial cell counter-receptor VCAM-1 delayed onset and reduced severity of the EAE. Furthermore, transfection of non-encephalitogenic clones to express α4 integrin restored the pathogenicity of the clones.

It is likely that induction of MMP-2 is at least one major role for α4 integrin in the disease process, as inhibitors of MMP-2 significantly delay the onset of disease (Graesser et al., 1998). Other investigators observed similar effects of MMP inhibitors on EAE disease progression in both mouse (Gijbels et al., 1994) and rat (Hewson et al., 1995) models. T cell clones transfected to overexpress pro-MMP-2 in the absence of α4β1 integrin expression elicit only modest transient clinical disease, while dually transfected cells that express both MMP-2 and α4β1 integrin are capable of eliciting typical, sustained EAE (Graesser, Mahooti and Madri, J. Neuroimmunol., In Press). mRNA and protein expression of MMP-2, MMP-7, MMP-8, and MMP-12 are also upregulated in brain lesions in a rat, delayed-type-hypersensitivity model of multiple sclerosis (Anthony et al., 1998). Together, these data suggest that regulation of expression and/or activation of MMPs plays a role in several models of T lymphocyte invasion into the CNS in vivo.

### A role in T cell transmigration in vitro?

While informative, many times animal models frequently do not lend themselves to mechanistic studies. We and others have utilized in vitro models in which T cells are co-cultured on a monolayer of endothelial cells and/or subendothelial matrix, mimicking the in vivo situation of blood vessel endothelium and subendothelial basement membrane. In this model, T cells transmigrate through the endothelial cell monolayer and subendothelial matrix, through a porous membrane and into a collection well. In these studies, both α4 integrin high-expressing and α4 integrin low-expressing T cell clones adhered well to both VCAM-1 positive and VCAM-1 negative endothelial cells (70% to 90%). However, high levels of T cell transmigration were observed only when T cells expressed significant levels of α4 integrin and endothelial cells expressed VCAM-1, suggesting that α4β1 integrin engagement is necessary for T cell transmigration (Graesser et al., 1998, Romanic and Madri, 1994). Furthermore, these studies suggest an inter-relationship between the presence and engagement of α4β1 and the induction and activation of MMP-2 during the process of T cell transmigration, because specific inhibitors of MMP-2 will block the high level of transmigration conferred by α4β1 integrin expression. Using similar in vitro models, other groups have also found that MMP inhibitors significantly reduce transmigration of lymphocytes (Leppert et al., 1995a, 1995b, 1996; Xia et al., 1996a).

Further evidence for the importance of integrin-mediated MMP regulation in T lymphocyte transmigration comes from studies with human peripheral blood T cells (Xia et al., 1996b). Treatment of CD4+ T cells with VIP (vasoactive intestinal peptide) leads to increased binding to fibronectin, secretion of MMPs, and migration through fibronectin-enriched matrigel. Neutralizing antibodies against α4 or β1 integrin did not directly suppress VIP-evoked MMP secretion, but did reduce degradation of type IV collagen by the T cells and subsequent migration. This result suggests that engagement of α4β1 integrin plays a role in regulating MMP activity, but not expression. This effect appears to be specific for α4β1 integrin, as neutralizing antibodies against α5 integrin reduced binding to fibronectin, but had no effect on the collagen-degrading activity of the T cells or their migration through matrigel in response to VIP. Another study has also connected MMP induction in human T cells, increased T cell transmigration in vitro and a possible role in CNS inflammation. Pretreatment of human T cells with interferon β1-b decreased interleukin-2 induced gelatinase production and gelatinase-dependent migration across a matrix by 90%. This study raises the possibility that the beneficial effects of interferon β1-b in multiple sclerosis patients may result from interference with the capacity of autoreactive T cells to migrate into the CNS (Yong et al., 1998).

**Organization of proteolytic complexes on T cell surfaces: A pre-requisite for transmigration?**

In several recent studies investigators have demonstrated the concentration of surface-bound proteinases on specific areas of migrating cells (Basbaum and Werb, 1996; Chen, 1996; Monsky et al., 1994; Nakahara et al., 1996) In recent studies we have observed a similar phenomenon in T cells which have adhered to and are initiating their migration through an endothelial cell monolayer and underlying matrix (Figure 1). Prior to adhesion to endothelial cell surfaces, α4β1 positive T cells express MT1-MMP and TIMP-2 on their surface in diffuse patterns. However, at early times following adhesion, α4β1 positive T cells are seen to express MMP-2 on their cell surface, co-localized with both MT1-MMP and TIMP-2. All three of the molecules in the "activation complex" are polarized to extended podosome regions on the migrating cells, as illustrated in Figure 1. We hypothesize that these regions will become the putative invading podosomes (invadopodiae) of the migrating T cells (Graesser, Mahooti and Madri, in preparation). These observations suggest a complex, multipoint control system for induction, secretion, surface tethering and activation of MMP-2 on the T cell surface. The generation, isolation and characterization of T cell clones having specific alterations in this migration pathway is likely to facilitate a more complete elucidation of this process in T cells as well as in other cell types. Thus, the cellular mechanism(s) mediating this process are currently undefined, but could involve activation and organization of the cytoskeleton, potentially triggered by specific integrin (α4β1 and/or other integrins) engagement and signaling as illustrated in Figure 1.

**Proteolysis of cell surface molecules: other roles for MMPs in inflammation?**

We assume infiltrating leukocytes utilize MMPs to degrade the components of the basement membrane, which presents a physical barrier to transmigration. MMPs may play many additional roles in during the inflammation process. MMPs are also capable of cleaving membrane-bound molecules, including cell surface adhesion molecules and cytokines. For example, MMP-7 (matrilysin) has been found to cleave β4 integrin from the surface of human prostate carcinoma cells (vonBredow et al., 1997). A variety of stimuli (chemoattractants, phorbol esters, L-selectin cross-linking) are known to simulate proteolytic cleavage of L-selectin ("shedding") from neutrophils and lymphocytes at a membrane-proximal extracellular domain (Jutila et al., 1989; Kishimoto et al., 1989). The physiologic L-selectin "sheddase" enzyme is believed to be a metalloproteinase, as shedding is completely prevented by hydroxymate-based metalloproteinase inhibitors (Preece et al., 1996; Bennett et al., 1996). In addition, L-selectin is susceptible to cleavage by known matrix metalloproteinases, MMP-1 and MMP-3. However, MMP-2 and MMP-9 have no effect, and L-selectin shedding is not inhibited by TIMPs. Shedding of L-selectin is thought to be an important part of the rolling mechanism, and L-selectin-mediated leukocyte rolling is slowed by preventing shedding (Hafezi-Maghadam and Ley, 1999; Walcheck et al., 1996). A metalloproteinase

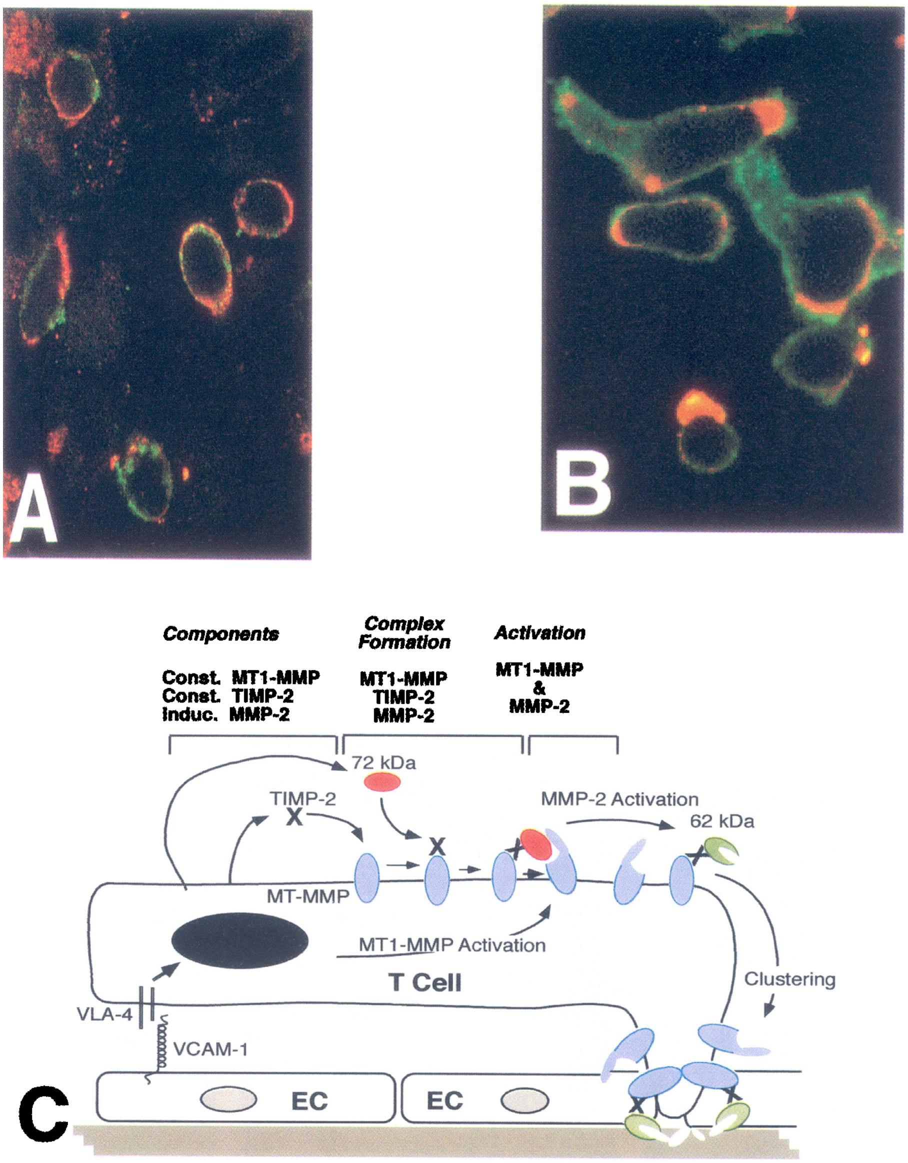

FIGURE 1 Representative Confocal microscopic micrograph of α4β1 high expressing T cells sixty minutes (A) and eight hours (B) after plating on a monolayer of VCAM-1 positive endothelial cells. A. The T cells (identified by Fluorescein labelled anti CD4) exhibit a diffuse peripheral plasma membrane localization of TIMP-2 (Rhodamine labelled) as well as MT1-MMP (not shown) before and after plating onto an endothelial cell monolayer. B. After two hours, the T cells have clustered TIMP-2 to specific membrane surface regions (Rhodamine labelled). This staining co-localizes with MT1-MMP and MMP-2 staining (data not shown), consistent with a tertiary complex formation. C. Schematic representation of the proposed α4β1-mediated induction, assembly and activation of MT-MMP, TIMP-2 and MMP-2 during T cell migration through the endothelium and its underlying basement membrane. Engagement of T cell α4β1 elicits the induction of pro-MMP-2, followed by clustering of MT-MMP and TIMP-2. This is followed by assembly of a tertiary complex composed of membrane-bound MT1-MMP and TIMP-2, functioning as a tethering complex mediating binding of pro-MMP-2. Following complex assembly, MT1-MMP activates the MMP-2 on the surface of the T cell, allowing for localized proteolysis of the ECM, facilitating migration of the T cell into the perivascular tissues (see Color Plate VIII at the back of this issue)

inhibitor has also been shown to block the phorbol-ester induced proteolytic cleavage of VCAM-1 from the cell surface of a set of $\gamma\delta$ T cells (Leca et al., 1995). MMPs may also play other roles in T cell immunological responses by acting on immunologically important molecules. For example, MMP-3 (stromelysin) as well as MMP-2 and MMP-9 process IL-1$\beta$ precursor into its biologically active form (Schonbeck et al., 1998). Inhibition of MMPs also block shedding of Fas ligand on T lymphocytes (Tanaka et al., 1998). The inter-play between adhesion molecules, MMPs and cytokines during an inflammatory responses appears to complex: adhesion through integrins and effects of cytokines act together to regulate MMP activity, and in response, adhesion molecules and cytokines are proteolytically cleaved by MMPs.

### CONCLUSION

The transition of T lymphocyte or other leukocytes from circulating cells to tissue resident cells is a complex process involving orchestrated engagement and disengagement of a variety of adhesion molecules and the initiation of several signaling cascades including those modulating the expression and affinity of cell surface integrins and the expression and activation of cell surface proteolytic enzymes (Figure 2). A more complete understanding of these processes will lead to the development of rational therapeutic approaches designed to beneficially modulate the inflammatory process.

### *References*

Albelda, S.M., Muller, W.A., Buck, C.A., Newman, P.J. (1991). Molecular and cellular properties of PECAM-1 (endo-CAM/CD31): a novel vascular cell-cell adhesion molecule. J. Cell Biol. **114**: 1059–1968.

Albelda, S.M., Oliver, P.D., Romer, L.H., Buck, C.A. (1990). Endo-CAM: a novel endothelial cell-cell adhesion molecule. J. Cell Biol. **110**: 1227–1237.

Alon, R., Rossiter, H., Springer, T.A., and Kupper, T.S. (1994). Distinct cell surface ligands mediate T lymphocyte attachment and rolling on P- and E- selectin under physiological flow. J. Cell Biol. **127**: 1485–1495.

Anthony, D.C., Ferguson, B., Matyzak, M.K., Miller, K.M., Esiri, M.M., and Perry, V.H. (1997). Differential matrix metalloproteinase expression in cases of multiple sclerosis and stroke. Neuropathol. Appl. Neurobiol. **23**: 406–415.

Anthony, D.C., Miller, K.M., Fearn, S., Townsend, M.J., Opdenaker, G., Wells, G.M., Clements, J.M., Chandler, S., Gearing, A.J., and Perry, V.H. (1998). Matrix metalloproteinase expression in an experimentally-induced DTH model of multiple sclerosis in the rat CNS. J. Neuroimmunol. **87**: 62–72.

Arner, E.C., and Tortorella, M.D. (1995). Signal transduction through chondrocyte integrin receptors induces matrix metalloproteinase synthesis and synergizes with interleukin-1. Arthritis Rheum. **38**: 1304–1314.

Arroyo, A.G., García-Pardo, A., Sánchez-Madrid, F. (1993). A high affinity conformational state on VLA integrin heterodimers induced by an anti-$\beta$1 chain monoclonal antibody. J. Biol. Chem. **268**: 9863–9868.

Azeh, I., Mader., M., Smirnov, A., Beuche, W., Nau, R., and Weber, F. (1998). Experimental pneumococcal meningitis in rabbits: the increase of matrix metalloproteinase-9 in cerebrospinal fluid correlates with leukocyte invasion.

Neurosci. Lett. **256**: 127–130.

Baron, J.L., Madri, J.A., Ruddle, N.H., Hashim, G., Janeway, C.A. (1993). Surface expression of $\alpha$4 integrin by CD4 T cells is required for their entry into brain parenchyma. J. Exp. Med. **177**: 57–68.

Basbaum, C.B. & Werb, Z. (1996). Focalized proteolysis: spatial and temporal regulation of extracellular matrix degradation at the cell surface. Curr. Opin. Cell Biol. **6**: 731–738.

Baumhueter, S., Singer, M.S., Henzel, W., Hemmerich, S., Renz, M., Rosen, S.D., and Lasky, L.A. (1993). Binding of L-selectin to the vascular sialomucin, CD34. Science **262**: 436–438.

Baumhueter, S., Dybdal, N., Kyle, C., and Lasky, L.A. (1994). Global vascular expression of murine CD34, a sialomucin-like endothelial ligand for L-selectin. Blood **84**: 2554–2565.

Bennet, T.A., Lynam, E.B., Sklar, L.A., and Rogelj, S. (1996). Hydroxamate-based metalloprotease inhibitor blocks shedding of L-selectin adhesion molecule from leukocytes; functional consequences for neutrophil aggregation. J. Immnol. **156**: 3093–3097.

Berg, E.L, Robinson, M.K., Watnock. R.A., and Butcher, E.C (1991). The human peripheral lymph node vascular addressin is a ligand for LECAM-1, the peripheral lymph node homing receptor. J. Cell Biol. **114**: 343–349.

Berlin, C., Berg., E.L., Briskin, M.J., Andrew, D.P., Kilshshaw, P.J., Holzmann, B., Weissman, I.L., Hamann, A., and Butcher, E.C. (1993). $\alpha 4\beta 7$ integrin mediates lymphocyte binding to the mucosal vascular addressin MAdCAM-1. Cell **74**: 185–195.

Berman, M.E. and Muller, W.A. (1995). Ligation of platelet/endothelial cell adhesion molecule 1 (PECAM-1/CD31) on monocytes and neutrophils increases binding capacity of leukocyte CR3 (CD11b/CD18). J. Immunol. **154**: 299–307.

Berman, M.E., Xie, Y., and Muller, W.A. (1996). Roles of platelet/endothelial cell adhesion molecule-1 (PECAM-1, CD31) in natural killer cell transendothelial migration and $\beta$2 integrin activation. J. Immunol. **156**: 1515–1524.

Bevilacqua, M.P. & Nelson, R.M. (1993). Selectins. J. Clin. Invest. **91**: 379–387.

Bevilacqua, M.P., Pober, J.S., Mendrick, D.L., Cotran, R.S., and Gimbrone, M.A. (1987). Identification of an inducible endothelial-leukocyte adhesion molecule, ELAM-1. Proc. Natl. Acad. Sci. USA. **84**: 9238–9242.

Bogen, S.A., Baldwin, H.S., Watkins, S.C., Albelda, S.A, and Abbas, A.K. (1992). Association of murine CD31 with transmigrating lymphocytes following antigenic stimulation. Am. J. Pathol. **141**: 843–854.

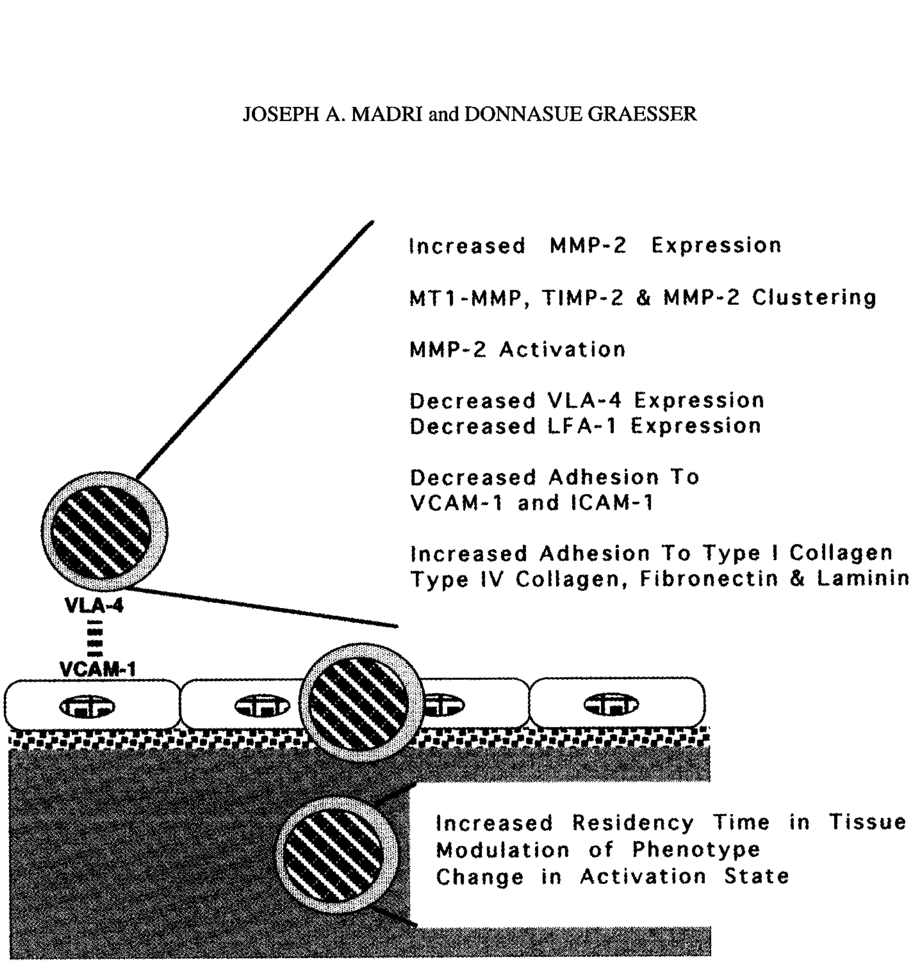

FIGURE 2 Schematic representation of the modulation of integrin expression, matrix degrading proteinases and matrix binding in T cells upon α4β1/VCAM-1 mediated adhesion to and transmigration through endothelial cells. Following engagement of α4β1 by endothelial cell VCAM-1, the T cell exhibits down-regulation of its expression of α4β1 and LFA-1 and upregulation of its expression of pro-MMP-2 (72 kDa gelatinase). This is followed by formation and clustering of a tertiary complex comprised of MT1-MMP, TIMP-2 and pro-MMP-2 and activation of MMP-2. In addition, these T cells exhibit reduced adhesion to VCAM-1 and ICAM-1 and increased adhesion to collagen types I and IV, fibronectin and laminin. Also noted were increases in endothelial cell uPA expression and activity and decreases in endothelial cell PAI-1 expression following engagement of VCAM-1 by α4β1. This figure was generated from data appearing in Graesser et al., 1998; Madri et al., 1996; Romanic et al., 1997 and Romanic & Madri, 1994

Butler, G., Butler, M., Atkinson, S., Will, H., Tamura, T., vanWestrum, S., Crabbe, T., Clements, J., d;Ortho, M. and Murphy, G. (1998). The TIMP-2 membrane type 1 metalloproteinase "receptor" regulates the concentration and efficient activation of progelatinase A: a kinetic study. J. Biol. Chem. **273**: 871–880.

Chan, B.M.C., Elices, M.J., Murpy, E., and Hemler, M.E. (1992). Adhesion to vascular cell adhesion molecule-1 and fibronectin: comparison of α4β1 (VLA-4) and α4β7 on the human B cell line JY. J. Biol. Chem. **267**: 8366–8370.

Chen, W.T. (1996). Proteases associated with invadopodia, and their role in degradation of extracellular matrix. Enzyme Protein **49**: 59–71.

Chintala, S.K., Sawaya, R., Gokaslan, Z.L., and Rao, J.S. (1996). Modulation of matrix metalloproteinase-2 and invasion in human glioma cells by α3β1 integrin. Cancer Lett. **103**: 201–208.

Christofidou-Solomidou., M., Nakada, N.T., Williams, J., Muller, W.A., and DeLisser, H.M. (1997). Neutrophil platelet endothelial cell adhesion molecule-1 participates in neutrophil recruitment at inflammatory sites and is down-regulated after leukocyte extravasation. J. Immunol. **158**: 4872–4878.

Davis, L.S., Oppenheimer-Marks, N., Bednarczyk, J.L., McIntyre, B.W., and Lipsky, P.E. (1990). Fibronectin promotes proliferation of naive and memory T cells by signaling through both the VLA-4 and VLA-5 integrin molecules. J. Immunol. **145**: 785–793.

deFougerolles, A.R. and Springer, T.A. (1992). Intercellular adhesion molecule –3, a third adhesion counter-receptor for lymphocyte function-associated molecule-1 on resting lymphocytes. J. Exp. Med. **175**: 185–190.

Diamond M.S. and Springer, T.A. (1994). The dynamic regulation of integrin adhesiveness. Curr. Biol. **4**: 506–517.

Diamond, M.S., Staunton, D.E., deFougerolles, A.R., Stacker, S.A., Garcia-Aguilar, J., Hibbs, M.L., and Springer, T.A. (1990). ICAM-1 (CD54): A counter-receptor for Mac-1 (CD 11b/Cd18). J. Cell Biol. **111**: 3129–3139.

Diamond, M.S., Staunton, D.E., Marlin, S.D., and Springer, T.A. (1991). Binding of the integrin Mac-1 (CD11b/CD18) to the third Ig-like domain of ICAM-1 (CD54) and its regulation by glycosylation. Cell **65**: 961–971.

Dustin, M.L. and Springer, T.A. (1989). T cell receptor cross-linking transiently stimulates adhesiveness through LFA-1. Nature **341**: 619–624.

Elices, M.J., Osborn, L., Takada, Y., Crouse, C., Luhowskyj, S., Hemler, M.E., Lobb, R.R. (1990). VCAM-1 on activated endothelium interacts with the leukocyte integrin VLA-4 at a site distinct from the VLA-4/fibronectin binding site. Cell 60: 577–584.

Fessler, L.I., Duncan, K.G., Fessler, J.H., Salo, T., Tryggvason, K. (1984). Characterization of the procollagen IV cleavage products produced by a specific tumor collagenase. J. Biol. Chem. **259**: 9783–9789.

Fina, L., Molgaard, H.V., Robertson, D., Bradley, N.J., Monaghan, P., Delia, D., Sutherland, D.R., Baker, M.A., and Greaves, M.F. (1990). Expression of the CD34 gene in vascular endothelial cells. Blood **75**: 2417–2426.

Gallatin, W.M., Weissman, I.L., and Butcher, E.C. (1983). A cell-surface molecule involved in organ-specific homing of lymphocytes. Nature **304**: 30–34.

Geng, J.G., Bevilacqua, M.P., Moore, K.L., McIntyre, T.M., Prescott, S.M., Kim, J.M., Bliss, G.A., Zimmerman, G.A., and McEver, R.P. (1990). Rapid neutrophil adhesion to activated endothelium mediated by GMP-140. Nature **343**: 30–34.

Gijbels, K., Galardy, R.E., Steinman, L., (1994). Reversal of experimental autoimmune encephalomyelitis with a hydroxamate inhibitor of matrix metalloproteinases. J. Clin. Invest. **94**: 2177–2182.

Gilles, C., Bassuk, J.A., Pulyaeva, H., Sage, E.H., Foidart, J.M., and Thompson, E.W. (1998). SPARC/osteonectin induces matrix metalloproteinase-2 activation in human breast cancer cell lines. Cancer Res. **58**: 5529–5536.

Goetzl, E.J., Banda, M.J., and Leppert. (1996). Matrix metalloproteinases in immunity. J. Immunol. **156**: 1–4.

Graesser, D., Mahooti, S., Haas, T., Davis, S., Clark, R.B., and Madri, J.A. (1998). The interrelationshipo of α4 integrin and matrix metalloproteinase-2 in the pathogenesis of experimental autoimmune encephalomyelitis. Lab. Invest. **78**: 1445–1458.

Haas, T.L., Davis, S.J., and Madri, J.A. (1998). Three-dimensional type-1 collagen lattices induce coordinate expression of matrix metalloproteinases MT1-MMP and MMP-2 in microvascular endothelial cells. J. Biol. Chem. **273**: 3604–3619.

Hafezi-Moghadam, A., and Ley, K. (1999). Relevance of L-selectin shedding for leukocyte rolling in vivo. J. Exp. Med. **189**: 939–947.

Hammer, D.A. and Apte, S.M. (1992). Simulation of cell rolling and adhesion on surfaces in shear flow: general results and analysis of selectin-mediated neutrophil adhesion. Biophys. J. **63**: 35–57.

Hauser, I., Setter, E., Bell, L., and Madri, J.A. (1993). Fibronectin expression correlates with U937 cell adhesion to migrating bovine aortic endothelial cells in vitro. Amer. J. Pathol. **143**: 173–179.

Hemler, M.E. (1990). VLA proteins in the integrin family: Structures, function, and their role on leukocytes. Annu. Rev. Immunol. **8**: 365–400.

Hewson, A., Smith, T., Leonard, J., and Cuzner, M. (1995). Suppression of experimental allergic encephalomyelitis in the Lew rat by the matrix metalloproteinase inhibitor R031–9790. Inflamm. Res. **44**: 345–349.

Hourihan, H., Allen, T.D., Ager, A. (1993). Lymphocyte migration across high endothelium is associated with increases in α4β1 integrin (VLA-4) affinity. J. Cell. Sci. **104**: 1049–1059.

Höyhtyä, M., Hujanen, E., Turpeeniemi-Hujanen, T., Thorgeirsson, U., Liotta, L., Tryggvason, K. (1990). Modulation of type IV collagenase activity and invasive behavior of metastatic human melanoma (A2058) cells in vitro by monoclonal antibodies to type IV collagenase. Int. J. Cancer **46**: 282–286.

Hu, M.C.T., Crowe, D.T., Weissman, I.L., and Holzmann, B. (1992). Cloning and expression of mouse integrin βp(β7): A functional role in Peyer's patch-specific lymphocyte homing. Proc. Natl. Acad. Sci. USA. **89**: 8254–8258.

Huhtala, P., Humphries, M., McDarthy, J., Tremble, P., Werb, Z., and Damsky, C. (1995). Cooperative signaling by α5β1 and α4β1 integrins regulates metalloproteinase gene expression in fibroblasts adhering to fibronectin. J. Cell Biol. **129**: 867–879.

Hynes, R.O. (1992). Integrins: Versatility, modulation, and signaling in cell adhesion. Cell **69**: 11–25.

Juliano, R.L., Haskill, S. (1993). Signal transduction from the extracellular matrix. J Cell Biol. **120**: 577–585.

Jutila, M., Torr, L., Berg, E., and Butcher, E. (1989). Function and regulation of the neutrophil MEL-14 antigen in vivo: comparison with LFA-1 and MAC-1. J. Immunol. **143**: 3318–3324.

Kansas, G.S., Wood, G.S., Fishwild, D.M., and Engleman, E.G. (1985). Functional characterization of human T lymphocyte subsets distinguished by monoclonal anti-leu-8. J. Immunol. **134**: 2995–3002.

Keszthelyi, E., Karlik, S., Hyduk, S., Rice, G., Gordon, G., Yednock, T., and Horner, H. (1996). Evidence for a prolonged role of α4 integrin throughout active experimental allergic encephalomyelitis. Neurology **47**: 1053–1059.

Kinoshita, T., Sato, H., Okada, A., Ohuchi, E., Imain, K., Okada, Y., and Seiki, M. (1998). TIMP-2 promotes activation of progelatinase A by membrane-type 1 matrix-metalloproteinase immobilized on agarose beads. J. Biol. Chem. **273**: 16098–16103.

Kishimoto, T., Jutila, M., Berg, E., and Butcher, E. (1989). Neutrophil Mac-1 and MEL-14 adhesion proteins inversely regulated by chemotactic factors. Science **245**: 1238–1241.

Kishimoto, T.K., Larson, R.S., Corbi, A.L., Dustin, M.L., Staunton, D.E., and Springer, T.A. (1989). The leukocyte integrins: LFA-1, Mac-1, and p150.95. Adv. Immunol. **46**: 149–182.

Kolb, S.A., Lahrtz, F., Paul, R., Leppert, D., Nadal, D., Pfistet, H..W., and Fontana, A. (1998). Matrix metalloproteinases and tissue inhibitors of metalloproteinases in viral meningitis: upregulation of MMP-9 and TIMP-1 in cerebrospinal fluid. J. Neuroimmunol. **84**: 143–150.

Kubota, S., Ito, H., Ishibashi, Y., and Seyama, Y. (1997). Anti-α3 integrin antibody induces the activated form of matrix metalloprotease-2 (MMP-2) with concomitant stimulation of invasion through matrigel by human rhabdomyosarcoma cells. Int. J. Cancer. **70**: 106–111.

Kuchroo, V., Sobel, R., Yamamura, T., Greenfield, E., Dorf M., and Lees, M. (1991). Induction of experimental allergic encephalomyelitis by myelin proteolipid protein specific clones and synthetic peptides. Pathobiol. **59**: 305–312.

Landis, R.C., Bennett, R.I., and Hogg, N. (1993). A novel LFA-1 activation epitope maps to the I domain. J. Cell Biol. **150**: 1519–1527.

Larsen, E., Celi, A., Gilbert, G.E., Furie, B.C., Erban, J.K., Bonfanti. R., Wagner, D.D., and Furie, B. (1989). PADGEM protein: a receptor that mediates the interaction of activated platelets with neutrophils and monocytes. Cell. **59**: 305–312.

Larsen, G.R., Sako, D., Ahern, T.J., Shaffer, M., Erban, J., Sajer, S.A., Gibson, R.M., Wagner, D.D., Furie, B.C., and Furie, B. (1992). P-selectin and E-selectin: Distinct but overlapping leukocyte ligand specificities. J. Biol. Chem. **267**: 11101–11110.

Lasky, L.A. (1992). Selectins: interpreters of cell-specific carbohydrate information during inflammation. Science. **258**: 964–969.

Lasky, L.A., Singer, M.S., Dowbenko, D., Imai, Y., Henzel, W.J., Grimley, C., Fennie, C., Gillet., N., Watson, S.R., and Rosen, S.D. (1992). An endothelial ligand for L-selectin is a novel mucin-like molecule. Cell. **69**: 927–938.

Lawrence, M.B. and Springer T. A. (1993). Neutrophils roll on E-selectin. J. Immunol. **151**: 6338–6346.

Lawrence, M.B. and Springer, T.A. (1991). Leukocytes roll on a selectin at physiologic flow rates: distinction from and prerequisite for adhesion through integrins. Cell. **68**: 859–873.

Leavesley, D. I., Oliver, J. M., Swart, B. W., Berndt, M. C., Haylock, D. N., Simmons, P. J. (1994). Signals from platelet/endothelial cell adhesion molecule enhance the adhesive activity of the very late antigen-4 integrin of human CD34+ hemapoietic progenitor cells. J. Immunol. **22**: 4073–4083.

Leca, G., Mansur, S.E., and Bensussan, A. (1995). Expression of VCAM-1 (CD106) by a subset of TCR gamma delta-bearing lymphocyte clones. Involvement of a metalloprotease in the specific hydrolytic release of the soluble isoform. J. Immunol. **154**: 1069–1077.

Leppert, D., Hauser, S.L., Kishiyama, J.L., An, S., Zeng, L., and Goetzl, E.J. (1995a). Stimulation of matrix-metalloproteinase-dependent migration of T cells by eicosanoids. FASEB J. **9**: 1473–1481.

Leppert. D., Waubant, E., Burk, M., Okensberg, J., and Hauser, S. (1996). Interferon β1-b inhibits gelatinase secretion and in vitro migration of human T cells; a possible mechanism for treatment efficacy in multiple sclerosis. Ann. Neurol. **40**: 846–852.

Leppert, D., Waubant, E., Galardy, R., Bunnet, N.W., Hauser, S.L. (1995b). T cell gelatinases mediate basement membrane transmigration in vitro. J. Immunol. **154**: 4379–4389.

Lewinsohn, D.M., Bargatze, R.F., and Butcher, E.C. (1987). Leukocyte-endothelial cell recognition: evidence of a common molecular mechanism shared by neutrophils, lymphocytes, and other leukocytes. J. Immunol. **138**: 4313–4321.

Liao, F., Huynh, H.K., Eiroa, A., Greene, T., Polizzi, E., and Muller, W.A. (1995). Migration of monocytes across endothelium and passage through extracellular matrix involve separate molecular domains of PECAM-1. J. Exp. Med. **182**: 1337–1343.

Liao, F., Ali, J., Greene, T., Muller, W.A. (1997). Soluble domain 1 of platelet-endothelial cell adhesion molecule (PECAM) is sufficient to block transendothelial migration in vitro and in vivo. J. Exp. Med. **185**: 1349–1357.

Liotta, L.A., S. Abe, P.G. Robey, Martin, G.R. (1979). Preferential digestion of basement membrane collagen by an enzyme derived from a metastatic murine tumor. Proc. Natl. Acad. Sci. USA **76**: 2268–2272.

Lo, S.K., Detmers, P.A., Levin, S.M., Wright, S.D. (1989). Transient adhesion of neutrophils to endothelium. J. Exp. Med. **169**: 1779–1793.

Lollo, B.A., Chan, K.W.H., Hanson, E.M., Moy, V.T., and Brian, A. A. (1993). Direct evidence for two affinity states for lymphocyte function-associated antigen-1 on activated T cells. J. Biol. Chem. **268**: 1–8.

Madri, J.A., Graesser, D., and Haas, T. (1996). The roles of adhesion molecules and proteinases in lymphocyte transendothelial migration. Biochem. Cell Biol. **74**: 749–757.

Madri, J.A., Pratt, B.M., Yannariello-Brown, J. (1988). Matrix driven cell size changes modulates aortic endothelial cell proliferation and sheet migration. Amer. J. Pathol. **132**: 8–27.

Marlin, S.D., and Springer, T.A. (1987) Purified intercellular adhesion molecule-1 is a ligand for lymphocyte function-associated antigen 1 (LFA-1). Cell **51**: 813–819.

McEver, R.P., Beckstead, J.H., Moore, K.L., Marshall-Carlson, L., and Bainton, D.F. (1989). GMP-140, a platelet alpha-granule membrane protein, is also synthesized by vascular endothelial cells and is localized in Weibel-Palade bodies. J. Clin. Invest. **84**: 92–99.

Mignatti, P., Robbins, E., Rifkin, D.B. (1986). Tumor invasion through the human amniotic membrane: requirement for a proteinase cascade. Cell **47**: 487–498.

Miller, M.D., and Krangel, M.S. (1992). Biology and biochemistry of the chemokines: a family of chemotactic and inflammatory cytokines. CRC Crit. Rev. Immunol. **12**: 12–17.

Monsky, W.L., Lin, C.Y., Aoyama, A., Kelly, T., Akiyama, S.K., Mueller, S.C., and Chen, W.T. (1994). A potential marker protease of invasiveness, seprase, is localized on invadopodia of human melanoma cells. Cancer Res. **54**: 5702–5710.

Montgomery, A.M.P., Sabzevari, H., and Reisfeld, R.A. (1993). Production and regulation of gelatinase B by human T cells. Biochim. Biophys. Acta. **1176**: 265–268.

Muller, W.A. (1995). The role of PECAM-1 in leukocyte emigration: studies in vitro and in vivo. J. Leukoc. Biol. **57**: 523–528.

Nakahara, H., Nomizu, M., Akiyama, S.K., Yamada, Y., Yeh, Y., Chen, W.T. (1996). A mechanism for regulation of melanoma invasion. Ligation of α6β1 integrin by laminin G peptides. J. Biol. Chem. **271**: 27221–27224.

Newman, P.J., Berndt, M.C., Gorski, J., White, G.C., Lyman, S., Paddock, C., Muller, W.A. (1990). PECAM-1 (CD31) cloning and relation to adhesion molecules of the immunoglobulin gene superfamily. Science **247**: 1219–1222.

Nojima, Y., Humphries, M.J., Mould, A.P., Yamada, K.M., Schlossman, S.F., Morimoto, C. (1990). VLA-4 mediates CD3-dependent CD4+ T cell activation via the CS1 alternatively spliced domain of fibronectin. J. Exp. Med. **172**: 1185–1192.

Nojima, Y., Rothstein, D.M., Sugita, K., Schlossman, S.F., Morimoto, C. (1992). Ligation of VLA-4 on T cells stimulates tyrosine phosphorylation of a 150 kDa protein. J. Exp. Med. **175**: 1045–1053.

Osborn, L., Hession, C., Tizard, R., Vassallo, C., Luhowskyj, S., Chi-Rosso, G., Lobb, R. (1989). Direct expression cloning of vascular cell adhesion molecule-1, a cytokine-induced endothelial protein that binds to lymphocytes. Cell. **59**: 1203–1211.

Preece, G., Murphy, G., and Ager, A. (1996). Metalloproteinase-mediated regulation of L-selectin levels on leucocytes. J. Biol. Chem. **271**: 11634–11640.

Remy, L., Bellaton, C., Pourreyron, C., Anderson, W., Pereira., A., Deumortier, J., and Jacquier, M.F. (1999). Integrins and metalloproteinases: an efficient collaboration in the invasive process. Bull. Cancer. **86**: 154–158.

Romanic, A.M., Graesser, D., Visintin, I., Baron, J.L., Janeway, C.A., and Madri, J.A. (1997). T cell adhesion to endothelial cell adhesion molecules and extracellular matrix is modulated upon transendothelial cell migration. Lab. Invest. **76**: 11–23.

Romanic, A.M., Madri, J.A. (1994). The induction of 72-kD gelatinase in T cells upon adhesion to endothelial cells is VCAM-1 dependent. J. Cell Biol. **125**: 1165–1178.

Rosen, S.D. (1993). Cell surface lectins in the immune system. Semin. Immunol. **5**: 237–247.

Rothlein, R., Dustin, M.L., Marlin, S.D., and Springer, T.A. (1986). A human intercellular adhesion molecule (ICAM-1) distinct from LFA-1. J. Immunol. **137**: 1270–1274.

Ruegg, C., Postigo, A.A., Sikorski, E.E., Butcher, E.C., Pytela, R., and Erle, D.J. (1992). Role of integrin α4β7/α4βp in lymphocyte adhesion to fibronectin and VCAM-1 and in homotypic cell clustering. J. Cell. Biol. **117**: 179–189.

Salo, T., Liotta, L., Tryggvason, K., (1983). Purification and characterization of a murine basement membrane collagen-degrading enzyme secreted by metastatic tumor cells. J. Biol. Chem. **258**: 3058–3063.

Sato, H., Takino, T., Okada, Y., Cao, J., Shinagawa, A., Yamamoto, E., Seiki, M. (1994). A matrix metalloproteinase expressed on the surface of invasive tumor cells. Nature. **370**: 6166.

Schonbeck, U., Mach, F., and Libby, P. (1998). Generation of biologically active IL-1 β by matrix metalloproteinases: a novel caspase-1-independent pathway of IL-1 β processing. J. Immunol. **161**: 3340–3346.

Sedlacek, R., Mauch, S., Kolb, B., Schatzlein, C., Eibel, H., Peter, H.H., Schmitt, J., and Krawinkel, U. (1998). Matrix metalloproteinase MMP-19 (RASI-1) is expressed on the surface of activated peripheral blood mononuclear cells and is detected as an autoantigen in rheumatoid arthritis. Immunobiol. **198**: 408–423.

Seftor, R.E.B., Seftor, E.A., Gehlsen, K.R., Stetler-Stevenson, W.G., Brown, P.D., Ruoslahti, E., Hendrix, M.J.C. (1992). Role of αvβ3 integrin in human melanoma cell invasion. Proc Natl Acad Sci USA **89**: 1557–1561.

Seftor, R.E.B., Seftor, E.A., Stetler-Stevenson, W.G., Hendrix, M.J.C. (1993). The 72 kDa type IV collagenase is modulated via differential expression of αvβ3 and α5β1 integrins during human melanoma cell invasion. Cancer Res **53**: 3411–3415.

Shimizu, Y. & Shaw, S. (1991). Lymphocyte interactions with extracellular matrix. FASEB J. **5**: 2292–2299.

Smith, C.W., Kishimoto, T.K., Abass, C., Hughes, B., Rothlein, R., McIntire, L.V., Butcher, E.C., and Anderson, D.C. (1991). Chemotactic factors regulate lectin adhesion molecule 1 (LECAM-1)-dependent neutrophil adhesion to cytokine-stimulated endothelial cells in vitro. J. Clin. Invest. **87**: 609–618.

Smith, C.W., Marlin, S.D., Rothlein, R., Toman, C., and Anderson, D.C. (1989). Cooperative interactions of LFA-1 and Mac-1 with intercellular adhesion molecule-1 in facilitating adherence and transendothelial migration of human neutrophils in vitro. J. Clin. Invest. **83**: 2008–2017.

Soilu-Hanninen, M., Roytta, M., Salmi, A., and Salonen, R. (1997). Therapy with antibody against leukocyte integrin VLA-4 (CD49d) is effective and safe in virus-facilitated experimental allergic encepahlomyelitis. J. Neuroimmunol. **72**: 95–105.

Springer, T. A. (1995a). Traffic signals on endothelium for lymphocyte recirculation and leukocyte emigration. Annu. Rev. Physiol. **57**: 827–872.

Springer, T. A. (1995b). Signals on endothelium for lymphocyte recirculation and leukocyte emigration: the area code paradigm. Harvey Lect. Series. **89**: 53–103.

Staunton, D.E., Marlin, S.D., Dustin, M.L., and Springer, T.A. (1989). Functional cloning of ICAM-2, a cell adhesion ligand for LFA-1 homologous to ICAM-1. Nature. **339**: 61–64.

Stetler-Stevenson, M., Mansoor, A., Lim, M., Fukushima, P., Kehrl, J., Marti, G., Ptaszynski, K., Wang, J., and Stetler-Stevenson, W.G. (1997). Expression of matrix metalloproteinases and tissue inhibitors of metalloproteinases in reactive and neoplastic lymphoid cells. Blood **89**: 1708–1715.

Streeter, P. R., Lakey-Berg, E., Rouse, B.T.N., Bargatze, R.F., and Butcher, E.C. (1988). A tissue-specific endothelial cell molecule involved in lymphocyte homing. Nature. **331**: 41–46.

Strongin, A.Y., Collier, I., Bannikov, G., Marmer, B.L., Grant, G.A., Goldberg, G.I., (1995). Mechanism of cell surface activation of 72-kDa Type IV collagenase. J. Biol. Chem. **270**: 5331–5336.

Tanaka, M., Itai, T., Adachi, M., and Nagata, S. (1998). Downregulation of Fas ligand by shedding. Nature Med. **4**: 31–36.

Tanaka, Y., Albelda, S.M., Horgan, K.J., van Seventer, G.A., Shimizu, Y., Newman, W., Hallam, J., Newman, P.J., Buck, C.A., Shaw, S. (1992). CD31 expressed on distinctive T cell subsets is a preferential amplifier of β1 integrin-mediated adhesion. J. Exp. Med. **176**: 245–253.

Thompson, E.W., Yu, M., Bueono, J., Jin, L., Maiti, S.N., Palao-Marco, F.L., Pulyaeva, H., Tamborlane, J.W., Tirgari, R., Wapnir, R. (1994). Collagen induced MMP-2 activation in human breast cancer. **31**: 357–370.

Tremble, P., Damsky, C., and Werb, Z. (1995). Components of the nuclear signaling cascade that regulate collagenase gene expression in response to integrin-driven signals. J. Cell Biol. **129**: 1707–1720.

Tremble, P., Chiquet-Ehrismann, R., and Werb, Z. (1994). The extracellular matrix ligands fibronectin and tenascin collaborate in regulating collagenase gene expression in fibroblasts. Mo. Biol. Cell. **5**: 439–453.

Vacca, A., Ribatti, D., Iurlaro, M., Albini, A., Minischetti, M., Bussolino, R., Pellegrino, A., Ria, R., Rusnati, M., Presta., M., Vincenti, V., Persico, M.G., and Damacco, F. (1998). Human lymphoblastoid cells produce extracellular matrix-degrading enzymes and induce endothelial cell proliferation, migration, morphogenesis, and angiogenesis. Int. J. Clin. Lab. Res. **28**: 55–68.

Vennegoor, C.J.G.M., van de Wiel-van Kemenade, E., Huijbens, R.J.F., Sanchez-Madrid, F., Melief, C.J.M., Figdor, C.G. (1992). Role of LFA-1 and VLA-4 in the adhesion of cloned normal and LFA-1 (CD11/CD18)-deficient T cells to cultured endothelial cells. J. Immunol. **148**: 1093–1101.

vonAndrian, U.H., Chambers, J.D., McEvoy, L.M., Bargatze, R.F., Arfors, K.E., and Butcher, E.C. (1991). Two-step model of leukocyte-endothelial cell interaction in inflammation: distinct roles for LECAM-1 and the β2 integrins in vivo. Proc. Natl. Acad. Sci. USA. **88**: 7538–7542.

vonBredow, D.C., Nagle, R.B., Bowden, G.T., and Cress, A.E. (1997). Cleavage of β4 integrin by matrilysin. Exp. Cell Res. **236**: 341–345.

Walcheck, B., Kahn, J., Fisher, J., Wang, B., Fisk, R., Payan, D., Feehan, C., Betageri, R., Darlak, K., Spatoloa, A., and Kishimoto, T. (1996). Neutrophil rolling altered by inhibition of L-selectin shedding in vitro. Nature **380**: 720–723.

Wayner, E.A., Garcia-Pardo, A., Humphries, M.J., McDonald, J.A., Carter, W.G. (1989). Identification and characterization of the T lymphocyte adhesion receptor for an alternative cell attachment domain (CS-1) in plasma fibronectin. J. Cell Biol. **109**: 1321–1330.

Wilkins, J.A., Stupack, D., Stewart, S., Ciaxia, S. (1991). β1 integrin-mediated lymphocyte adherence to extracellular matrix is enhanced by phorbol ester treatment. Eur. J. Immunol. **21**: 517–522.

Xia, M., Leppert, D., Hauser, S., Sreedharan, D., Nelson, P., and Krensky, A. (1996). Stimulus specificity of matrix metalloproteinase: dependence of human T cell migration through a model basement membrane. J. Immunol. **156**: 160–167.

Xia, M., Sreedharan, S.P., Dazin, P., Damsky, C.H., and Goetzl, E.J. (1996b). Integrin-dependent role of human T cell matrix metalloproteinase activity in chemotaxis through a model basement membrane. J. Cell Biochem. **61**: 452–458.

Yamada, A., Nikaido, T., Nojima, Y., Schlossman, S.F., Morimoto, C. (1991). Activation of human CD4 T lymphocytes: interaction of fibronectin with VLA-5 receptor on CD4 cells induces the AP-1 transcription factor. J. Immunol. **146**: 53–56.

Yednock, T.A., Cannon, C., Fritz, L.C., Sanchez-Madrid, F., Steinman, L., and Karin, N. (1992). Prevention of experimental autoimmune encephalomyelitis by antibodies against $\alpha4\beta1$ integrin. Nature **356**: 63–66.

Yong, V.W., Chabot, S., Stuve, O., and Williams, G. (1998). Interferon $\beta$ in the treatment of multiple sclerosis: mechanisms of action. Neurology **51**: 682–689.

